# Derivatisation-free FIA-MS/MS assay for rapid simultaneous screening of atypical myopathy biomarkers and acylcarnitine profiles from dried blood spots

**DOI:** 10.1007/s00216-026-06566-3

**Published:** 2026-05-27

**Authors:** Martina Kadláčková, Dana Dobešová, Eliška Ivanovová, Richard Masař, Petr Jahn, Eva Šamonilová, David Friedecký, Radana Brumarová

**Affiliations:** 1 Laboratory for Inherited Metabolic Disorders, Department of Medical Genetics, Faculty of Medicine and Dentistry, Palacký University Olomouc, Hněvotínská 3, 779 00 Olomouc, Czech Republic; 2 Laboratory for Inherited Metabolic Disorders, Department of Clinical Biochemistry, University Hospital Olomouc, Zdravotníků 248/7, 779 00 Olomouc, Czech Republic; 3 Equine Clinic, Faculty of Veterinary Medicine, University of Veterinary Sciences Brno, Palackého třída 1946/1, 612 00 Brno-Královo Pole, Czech Republic

**Keywords:** Flow injection analysis-tandem mass spectrometry, Dried blood spot sampling, Screening, Equine atypical myopathy, Hypoglycin A, MCPA-carnitine

## Abstract

Atypical myopathy (AM) is a frequently fatal, toxin-induced disease in horses, characterised by acute progression and mortality rates exceeding 70% within 72 h. The disease is caused by ingestion of seedlings or seeds of certain maple species that contain the toxins hypoglycin A (HGA) and methylenecyclopropylglycine (MCPrG). Early diagnosis depends on reliable detection of these compounds and their metabolites. However, most available LC-MS/MS assays are limited by derivatisation requirements and dependence on serum and urine matrices. Here, a rapid derivatisation-free flow injection analysis (FIA)-tandem mass spectrometry (MS/MS) method was developed for the simultaneous quantification of nine analytes: HGA, methylenecyclopropylacetyl-carnitine (MCPA-carnitine), and seven diagnostically relevant acylcarnitines, from dried blood spots (DBS). The procedure requires only 50 µL of whole blood, minimal sample preparation, and enables results within one minute. The assay provides low limits of quantification (0.01–0.1 μmol/L) with excellent linearity (*R*^2^ > 0.994), exhibiting analytical performance suitable for rapid screening of AM biomarkers. Moreover, analytes showed adequate stability in DBS during short-term storage at ambient temperature and prolonged storage at −20 °C. Clinical performance of the workflow was evaluated using 17 DBS samples from horses with AM and 15 control DBS samples. AM cases were defined based on the high specificity of HGA and MCPA-carnitine, with accompanying acylcarnitine profiles reflecting disease-associated metabolic disruption. Over 2 years, more than 250 DBS samples from horses suspected of AM were routinely analysed. Overall, the results establish a robust, high-throughput FIA-MS/MS platform integrating DBS sampling with non-derivatised analysis, offering a practical screening tool for remote sample collection, efficient transport, and multi-analyte diagnosis of AM.

**Graphical abstract:**

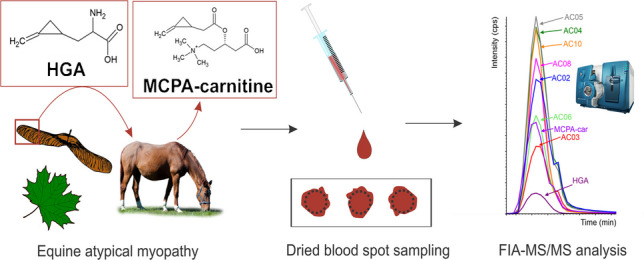

**Supplementary Information:**

The online version contains supplementary material available at 10.1007/s00216-026-06566-3.

## Introduction

Atypical myopathy (AM) is a severe and frequently fatal intoxication in grazing horses. It is characterised by acute non-exertional rhabdomyolysis [1] with mortality rates exceeding 70% within three days after the onset of clinical signs [2]. Affected animals typically present with muscle weakness, tremors, profuse sweating, and dark urine due to myoglobinuria, which often progresses rapidly to recumbency [3–5]. Because these clinical signs can resemble other acute equine disorders, particularly colic, there is a considerable risk of misdiagnosis in the field, potentially leading to inappropriate treatment [4]. This fulminant clinical course underscores the importance of rapid and reliable diagnostic tools.

The disease is caused by ingestion of seedlings or seeds of certain maple species, particularly *Acer pseudoplatanus* and *Acer negundo*, which contain the protoxins hypoglycin A (HGA) and methylenecyclopropylglycine (MCPrG) [6–9]. Following ingestion, these compounds are metabolised in skeletal muscle to methylenecyclopropylacetyl-CoA (MCPA-CoA) and methylenecyclopropylformyl-CoA (MCPF-CoA). These toxic metabolites inhibit multiple steps in fatty acid β-oxidation, resulting in secondary energy deficiency. During subsequent metabolism, MCPA-CoA and MCPF-CoA are converted to their carnitine and glycine conjugates, which are excreted in urine [10, 11]. Inhibition of β-oxidation also causes accumulation of short- and medium-chain acylcarnitines and acylglycines in blood and urine [12]. Together, these alterations provide complementary diagnostic information, while confirmation of AM usually relies on detecting protoxins or their metabolites in biological samples.

Liquid chromatography-tandem mass spectrometry (LC-MS/MS) has become the standard tool for quantifying HGA, MCPA-carnitine, and related metabolites. Many protocols use chemical derivatisation, including butylation [6, 13], dansylation [14], Fmoc derivatisation [15], or aTRAQ reagents [16], which can improve sensitivity, selectivity, and stability of analytes. However, derivatisation also increases cost, extends sample preparation time, and requires additional safety measures when handling reagents [17, 18]. These drawbacks limit the suitability of derivatised assays for emergency diagnostics, where speed and simplicity are essential.

Several derivatisation-free LC-MS/MS methods have been introduced in recent years. Rudolph et al. [19] reported a serum-based assay, but sensitivity was limited (LOD = 0.35 µmol/L for HGA and LOD = 0.01 µmol/L for MCPA-carnitine). González-Medina et al. [18] achieved higher sensitivity in the developed method for quantification of the toxins in equine serum and muscles and El-Khatib et al. [20] extended the approach to cow’s milk and urine. Although these studies demonstrate feasibility of derivatisation-free analysis, they generally focus on individual analytes and conventional matrices, leaving scope for methods that broaden analyte coverage directly from blood.

Dried blood spot (DBS) sampling provides a highly practical alternative to conventional liquid blood matrices such as serum or plasma. Whereas traditional blood collection typically requires several millilitres of venous blood, DBS necessitates only tens of microlitres [21]. Although blood is routinely obtained via venepuncture in veterinary practice, the minimal volume required means that a single drop from the syringe is sufficient for DBS preparation. Furthermore, by eliminating the need for immediate centrifugation or refrigeration, DBS represents an ideal strategy for in-field veterinary sampling. This eliminates the need for on-site laboratory equipment to separate serum or plasma, thus simplifying workflows for veterinary practitioners. Compared with conventional blood sampling, DBS offers improved logistical robustness, as analytes embedded in the dried matrix often remain stable for several days to weeks without the need for cold-chain transport [22]. Additionally, the dried nature of the samples eliminates the risk of spillage and substantially reduces the infectivity of certain viruses. This minimises biohazard risk, improving safety for handling personnel and facilitating cost-effective shipment to the laboratory via standard postal services [23]. Crucially, the desiccation process in DBS substantially reduces ex vivo enzymatic activity and cellular metabolism that typically persist in liquid whole blood. This immediate enzymatic inhibition helps limit analyte degradation and helps preserve the biochemical state of the sample at the time of collection [24]. Building upon its globally proven utility in newborn screening [25], DBS offers clear logistical and analytical advantages for veterinary diagnostics by streamlining both sample collection and transport under field conditions. Although recently published DBS-based LC-MS assays have addressed either acylcarnitines [26] or MCPA-carnitine [27], they were limited by the need for derivatisation, and none enabled simultaneous quantification of both toxins and acylcarnitines without chemical modification.

From an analytical perspective, currently available LC-MS/MS workflows for AM-related biomarkers remain limited in their suitability for rapid, high-throughput screening, owing to derivatisation requirements, chromatographic run times, and limited compatibility with DBS sampling.

In view of the above and the requirements of veterinarians, the aim of this study was therefore to develop a rapid, derivatisation-free FIA-MS/MS screening method for simultaneous quantification of HGA, MCPA-carnitine, and short- to medium-chain acylcarnitines from DBS. This proposed platform enables field-applicable, high-throughput screening of AM-related biomarkers with simple sample preparation and rapid analysis.

## Materials and methods

### Chemicals and reagents

Methanol (MeOH), water, and acetonitrile (ACN) were purchased from Honeywell Riedel-de Haën (Seelze, Germany). Formic acid (FA) was obtained from Thermo Fisher Scientific (Czech Republic). All chemicals were in LC-MS grade. A complete list of chemical standards (two toxins and seven acylcarnitines), including an internal standard (IS), is given in Supplementary Table S1.

### Biological material

For validation, a surrogate blood matrix (SBM) was prepared by pooling equal volumes of Li-heparinised whole blood from 15 healthy horses obtained during routine veterinary diagnostics. The pooled matrix was aliquoted (1 mL), and stored at −40 °C until analysis.

DBS were prepared from equine jugular blood spotted onto Whatman 903 cards. For clinical evaluation, 32 DBS samples were analysed: 17 from horses diagnosed with AM and 15 from healthy controls (Supplementary Table S2). Cards with DBS were stored at −20 °C until analysis. All samples originated from the Equine Clinic, Faculty of Veterinary Medicine, University of Veterinary Sciences Brno (Czech Republic).

### Standard preparation

#### Preparation of chemical standard stock solutions and internal standard

Acylcarnitine standards were prepared as 10 mmol/L stock solutions, with the HGA standard stock solution at a concentration of 6 mmol/L. All stock solutions were prepared in LC-MS water. The MCPA-carnitine stock solution was prepared in MeOH with a final concentration of 2 mmol/L. A solution of deuterated internal standard (IS) butyrylcarnitine-D_3_ (25 nmol/L) was prepared in MeOH. All stock solutions and aliquots of IS were stored at −80 °C.

#### Preparation of calibration standards

Calibration standards were prepared as a multicomponent mixture of the two toxins and seven acylcarnitines at the upper limit of quantification (ULOQ) concentration. The mixture was binary diluted with LC-MS grade water to obtain a ten-point calibration series (*n* = 3) down to the lower limit of quantification (LLOQ). Each level of the series was mixed 1:1 with SBM to generate matrix-matched calibrators. Aliquots (25 µL) were applied onto Whatman 903 cards, dried at room temperature for 1 h, and then processed as described in the Sample preparation section. The working ranges (LLOQ–ULOQ) are summarised in Supplementary Table S3. All calibration solutions were stored at −80 °C until use.

#### Preparation of quality control (QC)

Quality control (QC) samples were prepared as mixtures in LC-MS grade water at four concentration levels: LLOQ, LQC (low QC), MQC (medium QC), and HQC (high QC), covering the calibration range (Supplementary Table S4). These QC samples were used to assess method accuracy and precision, freeze-thaw stability and autosampler stability. To monitor stability of analytes in DBS, two levels of QC were prepared with analyte concentrations of 2 µmol/L (QC_1_) and 10 µmol/L (QC_2_). All QC stock solutions were stored at −80 °C until use. Final QC samples were generated by mixing each QC solution 1:1 with SBM to obtain matrix-matched samples. An aliquot (25 µL) of each mixture was spotted onto a Whatman 903 cards using a pipette and dried at room temperature for 1 h. Subsequent steps were performed as described in the Sample preparation section.

### Determination of endogenous concentration of analytes in surrogate blood matrix (SBM)

The endogenous concentrations of analytes in SBM, particularly acylcarnitines that occur physiologically in blood, were determined to enable appropriate correction during method validation. For this purpose, one unspiked DBS sample containing SBM and 10 DBS samples spiked with known concentrations of analytes covering the 10-point calibration range were prepared according to the procedure described in the Preparation of calibration standards section. 

For each spiked calibration level (*j*), the endogenous concentration of each analyte (*c*_*i,j*_) was calculated according to Eq. 1:

1 $${c}_{i,j}=\frac{{c}_{j}\times {P}_{0}}{{P}_{j}-{P}_{0}}$$

where *c*_*j*_ is the spiked concentration of the standard at calibration level *j*, *P*_*0*_ is the analyte/IS area ratio in the unspiked DBS sample, and *P*_*j*_ is the analyte/IS peak area ratio in the corresponding spiked DBS sample.

The final endogenous concentration (*c*_*i*_) was obtained as the mean of *c*_*i,j*_ values calculated across all 10 spiked calibration levels. This averaged endogenous concentration was subsequently used for correction of background contribution during calibration setup and validation experiments.

### Sample preparation

The DBS sample preparation procedure was optimised based on previously published protocols [13, 27]. Briefly, a 3.2-mm punch (Whatman 903) was extracted with 100 µL of methanol containing the internal standard (butyrylcarnitine-D_3_, final concentration 25 nmol/L), precipitated at −80 °C for 35 min and centrifuged (18,620 g, 10 min, 4 °C). The supernatant (85 µl) was lyophilised (−50 °C, 1 h), reconstituted in 100 µL of ACN-H_2_O (80:20, *v/v*) with 0.1% FA, and analysed by FIA-MS/MS.

### FIA-MS/MS analysis

FIA-MS/MS analysis was performed on a Triple Quad 6500 mass spectrometer (Sciex, Framingham, MA, USA) coupled to an UltiMate 3000 RS (Dionex, Sunnyvale, CA, USA). The instrument operated in positive electrospray ionisation mode, and data were acquired in multiple reaction monitoring (MRM). In this context, MRM transitions (precursor/product ions, declustering potential, entrance potential, collision energy and collision cell exit potential) were defined by automated MS/MS optimisation. Standard solutions (1 µmol/L in ACN-H₂O, 80:20, *v/v*, with 0.1% FA) were directly infused into the mass spectrometer. Candidate MRM transitions were evaluated for each analyte based on signal-to-noise (S/N) ratios. The most sensitive transitions were combined with optimised literature-based transitions to establish the final method. For each analyte, a quantifier transition was selected for routine quantification, while an additional qualifier transition was monitored to support analyte identification (Supplementary Table S5). The structures of precursor and product ions corresponding to the selected quantifier and qualifier transitions are illustrated in Supplementary Fig. S1. To further support analyte identification, ion ratios were calculated according to Eq. 2:

2 $$Ion\;ratio\left(\%\right)=\frac{A_{qualifier}}{A_{quantifier}}\times100$$

where *A*_*qualifier*_ and *A*_*quantifier*_ represent the peak areas of the qualifier and quantifier MRM transitions, respectively. Reference ion ratios were calculated as the mean ion ratio obtained from the six highest calibration levels analysed within the same batch, where signal intensities ensured stable and reproducible ion ratio values. Ion ratios obtained from DBS samples, QC samples and calibration standards were subsequently compared with these reference values and evaluated according to the acceptance criteria defined in Commission Decision 2002/657/EC [28]. The evaluated samples comprised DBS samples, QC samples (LQC and HQC) and calibration standards originating from multiple analytical batches.

The mobile phase consisted of ACN-H_2_O (80:20, *v/v*) with 0.1% FA and was delivered as a carrier solvent at a constant flow rate of 0.2 mL/min using the HPG 3400 pump module (Dionex, Sunnyvale, CA, USA). Samples were introduced into the continuous flow via the autosampler. To ensure stable flow conditions and sufficient backpressure, a capillary (length 5 m, internal diameter 0.1 mm) was installed in the flow path. The injection volume was 2 μL and the total run time per sample was 1 min. The optimised source parameters were: curtain gas 35 arb, collision gas 6 arb, ion spray voltage + 5500 V, source temperature 450 °C, and ion source gases 1 and 2 both set to 40 arb.

### Method validation

Validation parameters were assessed according to the guidelines of the European Medicines Agency (EMA) [29]. Validation parameters included calibration curve and range, accuracy, precision, carry-over, analyte stability in DBS samples, freeze-thaw stability, autosampler stability, and matrix effects. Because some of the investigated analytes (acylcarnitines) are endogenous metabolites physiologically present in blood, a true analyte-free matrix was not available. Therefore, endogenous concentrations in SBM were determined and considered during method validation.

#### Calibration curve and range

The calibration ranges of analytes were selected based on published physiological and pathological concentrations in horses [8, 12–15, 30, 31]. Each analytical run included a blank (Whatman 903 card without sample), a zero calibrator (SBM mixed 1:1 with H_2_O on Whatman 903 card), and ten calibration levels. Calibration performance was assessed across three independent analytical runs. With each run, calibration standards were injected consecutively in ascending concentration order from the lowest calibration level to the ULOQ level.

According to EMA guidelines [29], calibration curves should include at least six calibration points. The calibration range for each analyte was therefore defined from the initial ten-point calibration series as the interval where ≥ 6 levels met EMA acceptance criteria. Calibration curves were constructed from analyte/IS peak area ratios, with back-calculated concentrations required to be within ± 20% of nominal at LLOQ and ± 15% at higher levels.

For completeness, coefficients of determination (*R*^2^) exceeded 0.99 for all analytes. In addition, limits of detection (LOD) and quantification (LOQ) were estimated from S/N ratios at LLOQ levels within the calibration series (Eqs. 3 and 4). S/N ratios were determined using the S-To-N script implemented in Analyst software (Sciex). The analyte peak was manually selected and noise was calculated as peak-to-peak baseline noise measured in an adjacent region of equal width. LOD and LOQ values were reported only for descriptive purposes to facilitate comparison of analytical sensitivity with previously published LC-MS/MS methods, where these parameters are commonly reported.

3 $$LOD=\frac{3\times LLOQ}{\left(S/N\right)}$$

4 $$LOQ=\frac{10\times LLOQ}{(S/N)}=3.33\times LOD$$

#### Accuracy and precision

Within-run (*n* = 5) and between-run (*n* = 15) accuracy and precision were determined at four QC levels: LLOQ, LQC (3 × LLOQ), MQC (40% ULOQ), and HQC (75% ULOQ). QC samples were analysed in five replicates on three separate days, together with calibration standards. According to EMA guidelines [29], acceptance criteria required accuracy within 80–120% and precision (CV) ≤ 20% at the LLOQ, and accuracy within 85–115% and precision ≤ 15% at the other QC levels.

#### Carry-over

Carry-over was evaluated by analysing a blank sample prepared from an unspotted Whatman 903 card, processed identically to DBS samples, immediately following injection of a ULOQ calibration sample in three independent runs using the autosampler of the UltiMate 3000 RS system. The analytical run sequence consisted of QC samples, calibration standards injected in ascending order from the lowest concentration level to the highest calibration level (ULOQ), followed by a blank sample injection used for carry-over assessment. No additional flushing or intermediate conditioning steps were applied between these injections beyond the standard autosampler needle wash procedure with ACN-H_2_O (1:1, *v/v*). The blank sample was injected approximately 30 s after the ULOQ sample under identical instrumental conditions. Carry-over was considered negligible if the analyte response in the blank was < 20% of the analyte response at the LLOQ level and IS response was < 5% [29].

#### Stability of analytes in DBS samples

The stability was evaluated at two QCs, each containing all analytes. DBS (*n* = 3 per condition) were stored at room temperature and at −20 °C for 1, 2, 5, 7, 14, and 30 days; freshly prepared DBS (day 0, *n* = 3) served as reference. For each QC level, triplicate samples were analysed at each time point and storage condition. Stability was calculated as Eq. 5:

5 $$Stability \left(\%\right)=\frac{{c}_{t}}{{c}_{0}}\times 100$$

where *c*_*t*_ is the mean concentration at time point *t* (*n* = 3) and *c₀* is the mean concentration at the day 0 reference (*n* = 3).

Analytes were considered stable if Stability (%) remained within 85–115%, corresponding to ± 15% acceptance criterion.

#### Freeze-thaw stability

Freeze-thaw stability was assessed after three freeze-thaw cycles using LQC and HQC samples (*n* = 3). One cycle consisted of storage at −20 °C for 24 h followed by thawing at room temperature for 1 h. After the third cycle, samples were processed and analysed under the same conditions as freshly prepared DBS samples. Stability was expressed as the percentage of analyte concentration relative to freshly prepared DBS samples (t = 0) (Eq. 5). Analytes were considered stable when measured concentrations remained within ± 15% of the initial value, corresponding to 85–115% of the baseline concentration.

#### Autosampler stability

Autosampler stability was assessed by storing prepared DBS extracts in the autosampler at 4 °C and reanalysing them after 1 h, 2 h, and 24 h. Extracts prepared from DBS samples at LQC and HQC levels were compared with the initial measurement (time 0). Stability was considered acceptable if measured concentrations remained within 85–115% of the initial value (Eq. 5), corresponding to the ± 15% acceptance criterion.

#### Matrix effects

Matrix effects were assessed by comparing the peak areas of IS in DBS samples, calibration standards, and QC samples with those obtained from a blank extract prepared from an unspotted Whatman 903 card processed identically to DBS samples. The relative IS response in matrix-containing samples was expressed as a percentage of the response measured in the blank extract and used as an indicator of matrix-induced signal suppression or enhancement.

### Routine performance monitoring

Method performance during routine analysis was periodically verified using in-house QC samples at two concentration levels. QC samples were analysed together with calibration standards and DBS study samples to confirm appropriate method performance.

### Data analysis

MS/MS acquisition was controlled using Analyst software (version 1.6.2; Sciex, Framingham, MA, USA), data were processed in SciexOS (version 2.0.1; Sciex), and results were visualised using GraphPad Prism (version 9.4.1; GraphPad Software, San Diego, CA, USA).

## Results

### FIA-MS/MS analysis

**Fig. 1:**
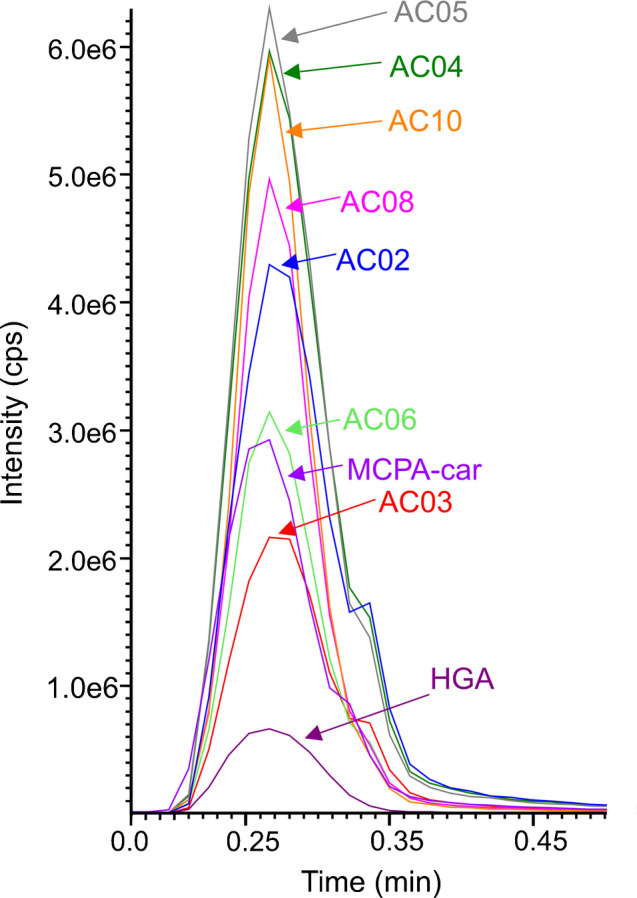
Representative FIA-MS/MS profile of a ULOQ calibration sample acquired in positive ionisation mode, showing the nine target analytes (HGA, MCPA-carnitine, and seven acylcarnitines)

The FIA-MS/MS method was developed for the quantification of nine diagnostically relevant analytes in equine AM consisting of HGA, MCPA-carnitine, and seven acylcarnitines (acetylcarnitine, AC02; propionylcarnitine, AC03; butyrylcarnitine/isobutyrylcarnitine, AC04; valerylcarnitine/isovalerylcarnitine/2-methylbutyrylcarnitine, AC05; hexanoylcarnitine, AC06; octanoylcarnitine, AC08; and decanoylcarnitine, AC10). All MS/MS parameters are listed in Supplementary Table S5. A representative extracted ion chromatogram of standards in ULOQ is shown in Fig. 1.

Ion ratio evaluation confirmed the consistency of the selected MRM transitions. Reference ion ratios derived from calibration standards were compared with those obtained from DBS samples (*n* = 8), QC samples and LQC and HQC levels (*n* = 15) and calibration standards (*n* = 10). For all analytes, ion ratios remained within the acceptable tolerance limits defined in Commission Decision 2002/657/EC for MRM-based identification. Reference ion ratio values and the corresponding acceptance ranges are summarised in Supplementary Table S6, while the distribution of ion ratios across calibration standards, QC samples and DBS samples is shown in Supplementary Figure S2.

### Method validation

#### Determination of endogenous concentration of analytes in surrogate blood matrix (SBM)

For method validation, the calibration curve and QC samples were prepared by 1:1 dilution with SBM. To ensure accurate quantification, endogenous analyte concentrations in SBM were determined (Supplementary Table S7).

#### Calibration curve and range

**Fig. 2:**
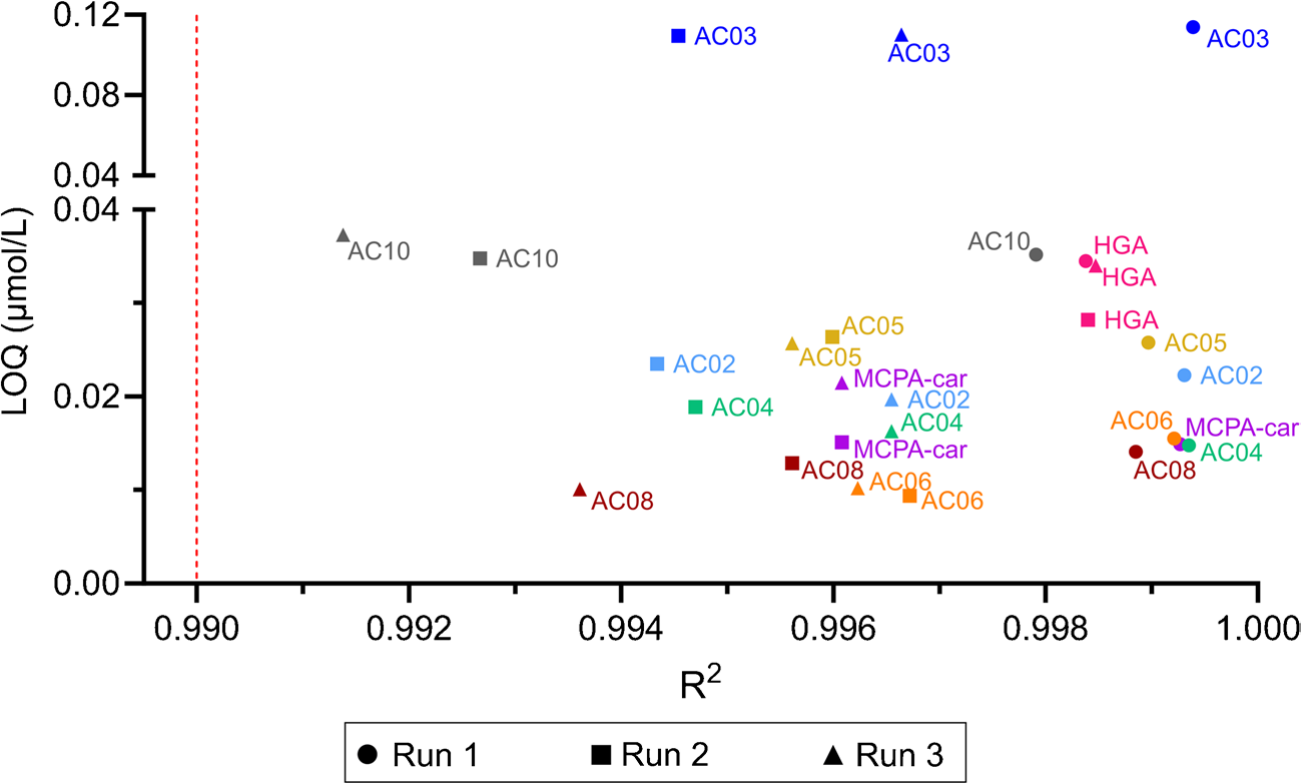
Relationship between coefficients of determination (*R*^2^) and limits of quantifications (LOQ) for all analytes. The red dotted line indicates the acceptance criterion (*R*^2^ ≥ 0.99), with each point corresponding to one of three independent validation runs (*n* = 3); points are not displayed in acquisition order

All nine analytes demonstrated linear responses within their respective calibration ranges, with mean *R*^2^ > 0.994 (Fig. 2). For the key biomarkers of AM, HGA and MCPA-carnitine, *R*^2^ were 0.998 and 0.997 (LOQ = 0.03 µmol/L and 0.02 µmol/L). A slight variation in *R*^2^ values between individual validation runs was observed, which is likely attributable to minor variations in instrument performance between runs. Among the evaluated regression models, linear regression with a 1/x weighting factor was selected due to the wide concentration range of the calibration standards (approximately two to three orders of magnitude), as it improved the fit at lower concentration levels while maintaining consistent calibration performance across the entire working range. The calibration curves of all analytes contained at least six calibration points. Detailed parameters of calibration curve, regression parameters, LODs, LOQs, and *R*^2^ values are provided in Supplementary Table S8 and Supplementary Fig. S3.

#### Accuracy and precision

Intra-day accuracy (%) and precision (CV, %) are summarised in Supplementary Table S9 and shown in Supplementary Fig. S4. At MQC and HQC levels, all analytes met the acceptance criteria. At lower concentration levels (LLOQ and LQC), several analytes showed minor deviations in accuracy and precision, most frequently among acylcarnitines with shorter chain lengths (AC02–AC06). AC08 and AC10 exceeded accuracy limits in every run (120.8–144.1%) but maintained acceptable precision. Intra-day performance was therefore reliable at medium and high concentrations, while reproducibility decreased at low levels.

**Fig. 3:**
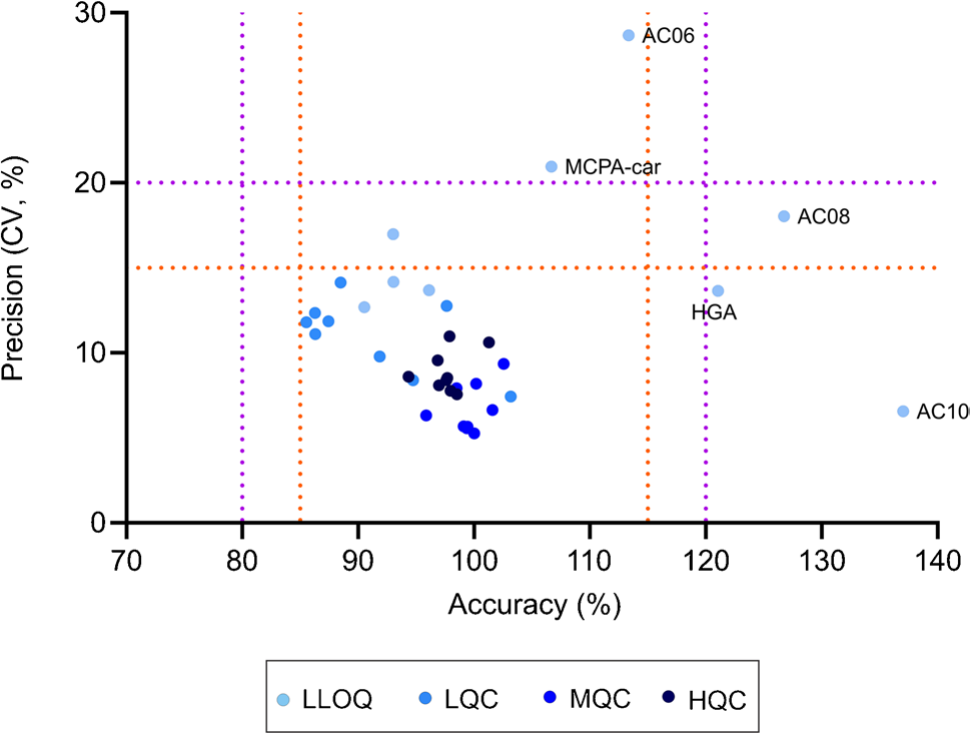
Inter-day accuracy and precision (*n* = 15) with acceptance criteria indicated by purple lines for LLOQ and orange lines for LQC, MQC, and HQC. Only analytes that exceeded the acceptance limits are labelled

Inter-day results are presented in Fig. 3 and detailed in Supplementary Table S10. Accuracy and precision remained within acceptance criteria across all levels, with occasional greater variability at the LLOQ (HGA, MCPA-carnitine, AC06, AC08, AC10).

#### Carry-over

Carry-over was negligible for all analytes, with analyte responses in blank samples (*n* = 3) consistently below 20% of the corresponding LLOQ signals for analytes and below 5% of the LLOQ response for IS (Supplementary Table S11).

#### Stability

**Fig. 4:**
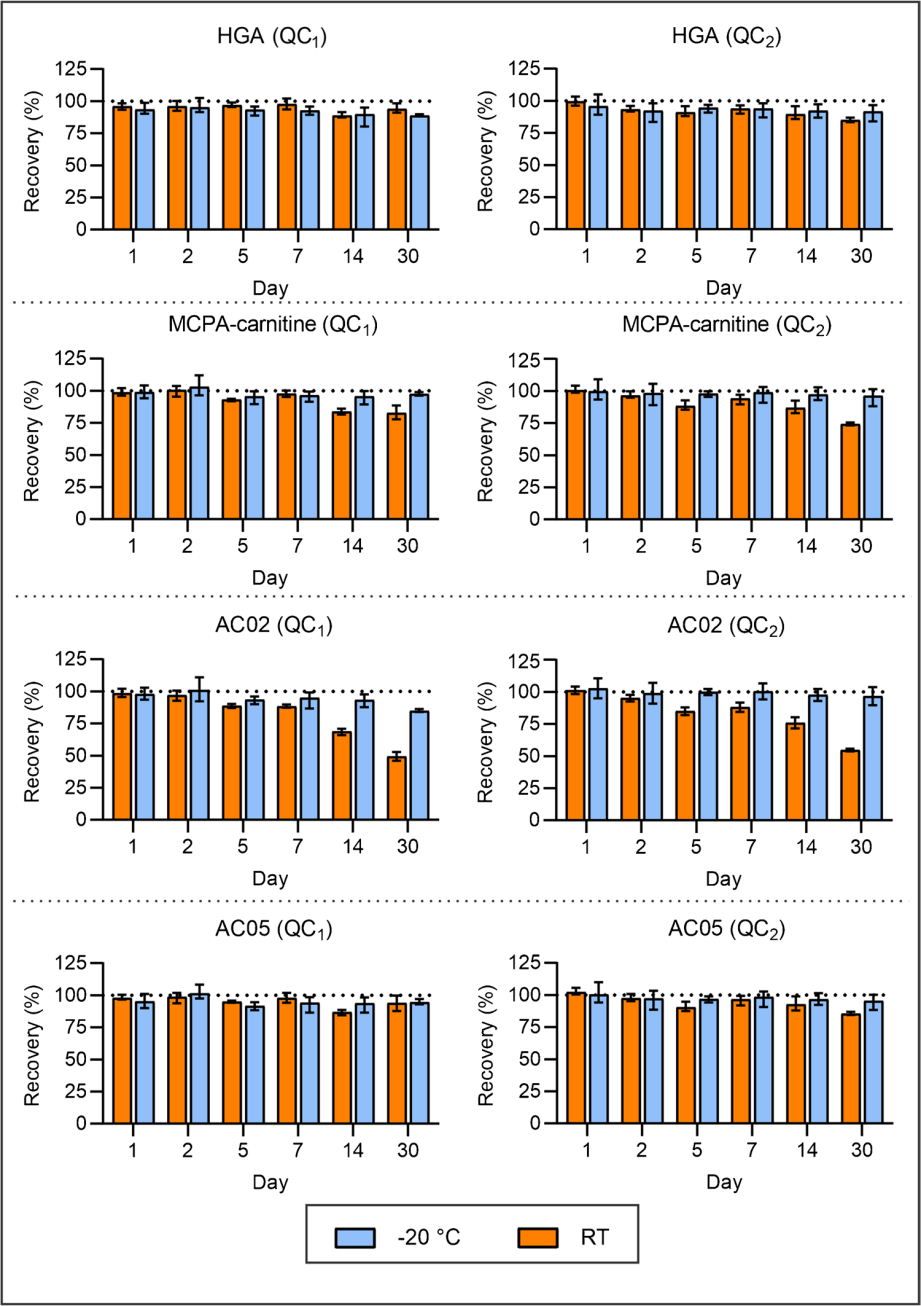
Stability of hypoglycin A (HGA), MCPA-carnitine, acetylcarnitine (AC02) and valerylcarnitine/isovalerylcarnitine/2-methylbutyrylcarnitine (AC05) in the DBS sample (QC_1_ = 2 µmol/L; QC_2_ = 10 µmol/L) after 1, 2, 5, 7, 14, and 30 days. DBS samples were stored at −20 °C and room temperature (RT)

The stability of analytes in DBS samples was evaluated over 30 days at room temperature and at −20 °C at two QC levels. Results are summarised in Supplementary Tables S12–S13 and illustrated in Fig. 4. At room temperature, most analytes were stable for up to 7 days, but degradation occurred thereafter, particularly for MCPA-carnitine, AC02, and AC03. By day 14, recoveries for these compounds at QC_1_ fell below 85%, and after 30 days, AC02 showed the most pronounced loss (≈ 50% of baseline). The remaining analytes retained acceptable stability at both QC levels. At −20 °C, all analytes remained within acceptance criteria, except AC02, which showed a marginal decline to 84.8% after 30 days (QC_1_). Overall, DBS samples were stable for at least one week at room temperature and up to 30 days when stored at −20 °C.

Freeze-thaw stability was evaluated after three freeze-thaw cycles at LQC and HQC levels. After three cycles, stability values ranged from 88.2 to 97.2% at the LQC level and 90.7 to 108.8% at the HQC level, confirming that repeated freezing and thawing did not significantly affect analyte concentrations in DBS samples. Detailed results are summarised in Supplementary Table S14.

Autosampler stability of processed samples was evaluated at LQC and HQC levels after storage at 4 °C for up to 24 h. All analytes remained stable during the tested period. The observed stability values ranged from 99.5 to 111.4% at the LQC level and 96.5 to 104.4% at the HQC level relative to the initial values (*t* = 0). Detailed results are provided in Supplementary Table S15.

#### Matrix effects

Matrix effects were evaluated across all analysed samples, including study samples, calibration standards, and QC samples (*n* = 159) measured across multiple analytical runs. The mean relative internal standard response in matrix-containing samples was 65.2 ± 2.2%, corresponding to a coefficient of variation of 3.4%, indicating moderate ion suppression of approximately 35%. The low variability demonstrates consistent matrix effects across different sample types and analytical runs.

### Method application

**Fig. 5:**
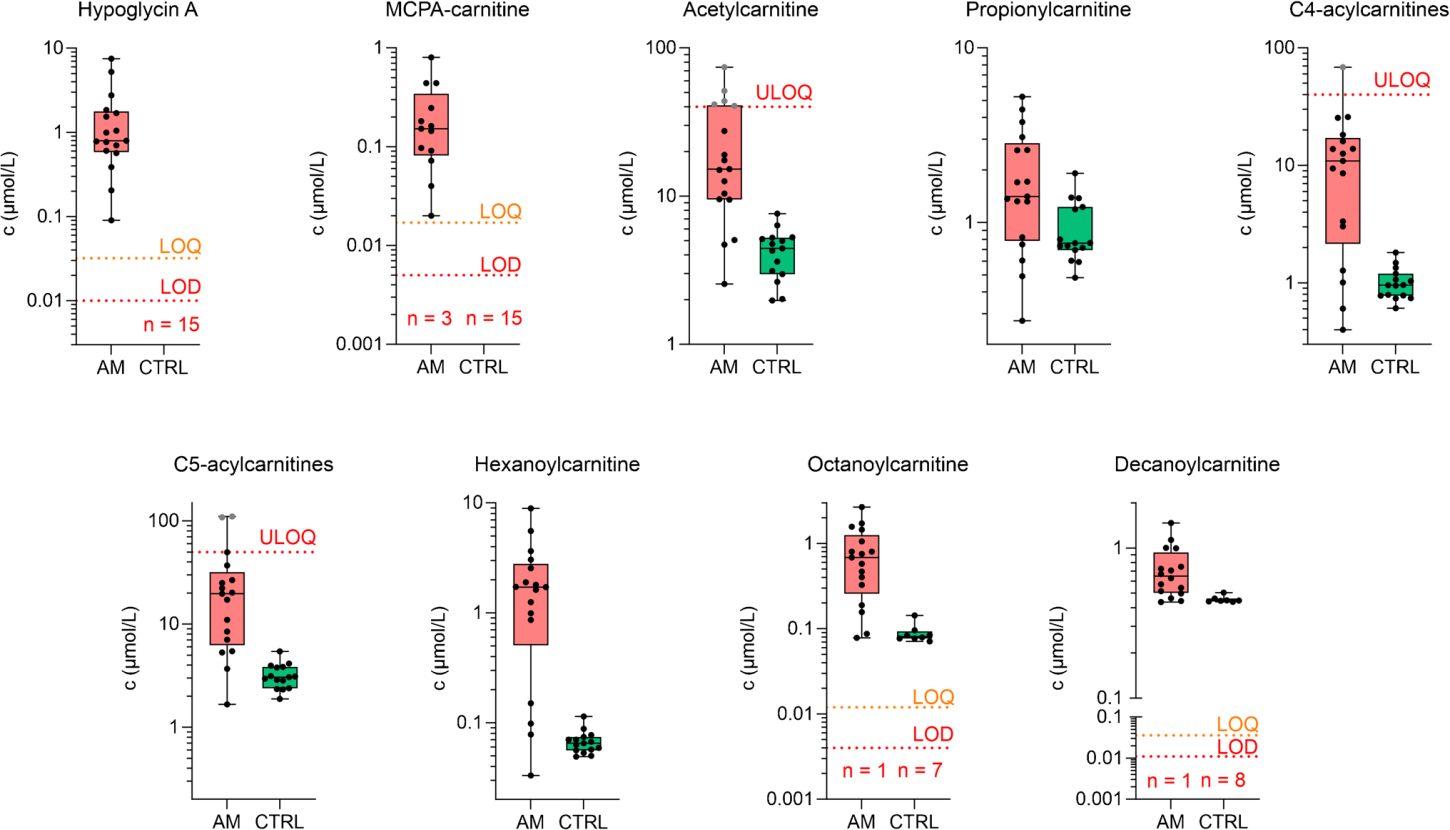
Distribution of hypoglycin A (HGA), MCPA-carnitine, and acylcarnitines in DBS samples from AM-affected horses (AM; *n* = 17) and healthy controls (CTRL; *n* = 15). Boxplots display the median and interquartile range; whiskers indicate minimum and maximum values. Dashed lines indicate analytical limits, where applicable: orange lines represent the limit of quantification (LOQ), red lines represent the limit of detection (LOD), and the upper limit of quantification (ULOQ) is indicated where relevant. Samples exceeding the ULOQ are shown in grey

The developed FIA-MS/MS method was applied to 32 clinical DBS samples, comprising 17 from horses diagnosed with AM and 15 from healthy controls. HGA and/or MCPA-carnitine were detected exclusively in AM cases, confirming their diagnostic specificity. The distribution of HGA, MCPA-carnitine, and representative acylcarnitines in both studied groups is shown in Fig. 5. Measured concentration ranges (including median, minimum and maximum values) of analytes in DBS samples from affected and healthy horses are summarised in Supplementary Table S16. For samples exceeding the ULOQ level, measured responses were retained for visualisation in Fig. 5 and are indicated in grey. These values should be interpreted as semi-quantitative estimates. Collectively, these data demonstrate that DBS sampling combined with FIA-MS/MS analysis enables clear differentiation between AM-affected and healthy controls.

## Discussion

AM presents a diagnostic challenge in equine practice due to its clinical overlap with gastrointestinal colic and other types of myopathies [32]. Early recognition is essential, as management strategies differ markedly; exercise and mobilisation beneficial in colic can accelerate rhabdomyolysis and lead to fatal outcomes in AM. Rapid and reliable differential diagnosis is therefore critical for appropriate therapeutic intervention and effective outbreak control.

Here we present a novel derivatisation-free FIA-MS/MS method for the simultaneous quantification of HGA, MCPA-carnitine, and seven diagnostically relevant short- and medium-chain acylcarnitines from DBS samples for AM diagnosis. To our knowledge, this is the first approach enabling parallel detection of AM protoxin/toxin and associated acylcarnitine alterations directly from DBS, offering advantages in analytical scope and logistical feasibility for field application.

Several analytical approaches have been reported for the determination of HGA, MCPA-carnitine and related biomarkers used for the detection of equine AM or HGA intoxication in humans [33, 34]. However, these methods differ substantially in sample preparation strategies, analyte coverage, and analytical throughput (Supplementary Table S17). Most reported assays rely on chromatographic LC-MS/MS workflows applied to serum or plasma, with instrumental analysis times typically ranging from 7 to 18 min per sample and detection limits frequently reaching the low nmol/L range [6], 13–16, 18–19,, 33–36]. In terms of analytical performance, these methods typically achieve accuracy and precision within ± 15%, whereas slightly increased variability observed in the present method, particularly at low concentration levels, reflects the trade-off associated with the derivatisation-free FIA-MS/MS screening, while still allowing reliable differentiation between affected and non-affected individuals. Although most studies focus on blood-derived matrices, HGA, MCPrG and their metabolites have also been determined in urine [6, 8, 13, 15, 18, 35] and less conventional matrices such as milk [20, 37], muscle [18] and tissues [11] or equine hair [38]. Direct comparison of analytical performance should therefore be interpreted cautiously, as most published methods analyse liquid matrices, whereas the present approach is based on DBS extraction, which inherently involves a higher dilution factor. Sander et al. [26] and Karlíková et al. [27] demonstrated the applicability of dried matrix approaches for the analysis of AM-related toxins and acylcarnitines. However, these workflows relied on derivatisation and did not enable simultaneous determination of toxins and acylcarnitine profiles within a single analytical run. In addition, the derivatisation-based workflow reported by Karlíková et al. [27] required approximately 24 h of sample preparation, which substantially limited analytical throughput. To our knowledge, this study presents the first derivatisation-free FIA-MS/MS workflow enabling simultaneous determination of HGA, MCPA-carnitine and diagnostically relevant acylcarnitines from dried blood spots (~ 50 µL blood) within approximately 1 min per sample. Although the achieved LOQs (HGA 0.03 µmol/L, MCPA-carnitine 0.02 µmol/L, acylcarnitines 0.01–0.1 µmol/L) are higher than those reported for some chromatographic assays, the sensitivity remains fully adequate for screening purposes, as biomarker concentrations in AM-affected animals typically exceed these limits by orders of magnitude. Importantly, the method was primarily designed as a rapid screening tool, prioritising analytical throughput, simplified sample preparation, and simultaneous detection of multiple biomarkers. Consequently, the proposed workflow represents a practical compromise between analytical sensitivity and throughput, while the DBS format simplifies sample collection, transport and storage for rapid AM diagnostics under field conditions.

The analytical performance indicates that this simplified workflow provides sufficient quantitative reliability for rapid screening applications. A single isotopically labelled IS (butyrylcarnitine-D_3_) was used as a representative acylcarnitine analogue to correct variability during sample preparation and ionisation. This compound is widely used as an internal standard in FIA-MS/MS acylcarnitine profiling in newborn screening programmes [39]. A potential limitation of this approach is that isotopically labelled analogues were not available for all analytes included in the panel; however, the overall validation results indicate that butyrylcarnitine-D_3_ provided adequate correction across the analysed compounds. Ion ratio evaluation further supported reliable analyte identification, as all measured ion ratios remained within the acceptance ranges defined in Commission Decision 2002/657/EC [28]. Validation parameters were assessed in accordance with the EMA guideline for the intended screening application [29]. Calibration curves covered both physiological and pathological levels of analytes, and sensitivity was sufficient to quantify clinically relevant levels of AM biomarkers and acylcarnitines. Accuracy and precision met acceptance criteria at MQC and HQC, while greater variability was observed at the lowest levels (HGA, MCPA-carnitine, AC06, AC08, and AC10). Such behaviour near the LLOQ is commonly associated with reduced S/N ratios and for these analytes at the lowest concentration range the method should be interpreted as semi-quantitative and screening-oriented rather than fully quantitative. Stability assessment confirmed the suitability of DBS sampling, with all analytes remaining within acceptable limits for 30 days at −20 °C and up to seven days at room temperature before gradual loss of MCPA-carnitine, AC02, and AC03 became evident. In addition, analytes remained stable during repeated freeze-thaw cycles and in processed extracts stored in the autosampler. These findings highlight the robustness of DBS-based analysis and demonstrate that DBS sampling supports both short-term ambient transport and long-term frozen storage, while also providing sufficient stability during routine laboratory workflows involving repeated freezing and thawing or delayed autosampler analysis. Importantly, the observed ion suppression was highly reproducible across analysed samples, indicating a stable matrix influence. Such consistent matrix effects can be reliably compensated by the use of isotopically labelled internal standards together with matrix-matched calibration prepared in SBM-based DBS samples. Because calibrators, QC samples and study samples underwent identical sample preparation and extraction procedures, matrix effects did not adversely affect the quantitative performance or robustness of the method. Recovery was not assessed as a standalone parameter, since all calibrators, quality controls, and study samples were processed identically. In addition, selectivity assessment using blank matrix samples was not feasible, as several analytes (acylcarnitines) are present endogenously in blood. Within this DBS-based workflow, accuracy and precision were considered the most appropriate indicators of overall analytical performance.

Application to clinical samples confirmed the diagnostic specificity of HGA and MCPA-carnitine, which were present exclusively in AM-affected horses. Affected animals also showed elevated short- and medium-chain acylcarnitines, reflecting inhibition of mitochondrial fatty acid oxidation. These results support the diagnostic relevance of combining toxin detection with acylcarnitine profiling, consistent with previous reports [6, 10, 12, 13, 26, 30, 31, 40, 41].

In addition to HGA and MCPA-carnitine, MCPrG was evaluated during method development. However, its determination was not feasible within the derivatisation-free FIA-MS/MS workflow due to insufficient sensitivity in DBS samples. This is consistent with its lower physiological concentration compared to HGA [8, 14, 15], as well as its generally lower abundance in plant material [42]. Although analytical methods for MCPrG determination have been reported in human biological matrices [33, 34, 36, 43] and in animal matrices such as milk or urine [20, 37], these approaches rely on chromatographic separation and/or derivatisation to achieve sufficient sensitivity.

While the developed FIA-MS/MS method enables rapid and practical screening, certain limitations should be noted. The FIA-MS/MS does not allow for the resolution of isomeric acylcarnitines such as AC04 (butyrylcarnitine/isobutyrylcarnitine) and AC05 (valerylcarnitine/isovalerylcarnitine/2-methylbutyrylcarnitine), as compounds with identical molecular weights share the same MRM transitions and are therefore quantified together. In addition, some AM-related metabolites (MCPF-carnitine, MCPA-glycine, and MCPF-glycine) were not included in the method due to the lack of commercial standards.

Overall, the proposed workflow combines rapid high-throughput analysis with simplified sampling logistics, providing a practical analytical tool for rapid screening and confirmation of atypical myopathy under field veterinary conditions.

## Conclusion

This study presents the first derivatisation-free FIA-MS/MS method enabling rapid screening of AM biomarkers and acylcarnitines from DBS. By combining minimal sample volume with a one-minute analysis time, the method provides a practical tool for diagnostic screening. The demonstrated stability of DBS samples makes the developed method suitable for field sampling, ambient transport and routine diagnostics workflow. By enabling rapid confirmation of suspected outbreaks, the method provides veterinarians with a reliable decision-making tool for timely diagnosis and appropriate clinical management of affected horses. The approach also offers flexibility for future expansion to include additional AM-related toxins as reference materials become available.

## Supplementary Information

Below is the link to the electronic supplementary material.

Supplementary file1 (XLSX 143 KB)

Supplementary file2 (DOCX 1.03 MB)

## Data Availability

The datasets generated during and/or analysed during the current study are available from the corresponding author on reasonable request.

## Declarations

### Source of biological material

Blood samples were obtained from horses during routine veterinary diagnostic procedures and were used for the purposes of this study.

### Animal welfare

All procedures were part of routine veterinary practice, and no experimental interventions affecting animal welfare were performed. Informed consent was obtained from the animal owners.
