# Supplementary material for: Derivatisation-free FIA-MS/MS assay for rapid simultaneous screening of atypical myopathy biomarkers and acylcarnitine profiles from dried blood spots
Source: Anal Bioanal Chem. 2026 May 27;418(14):4637–49. doi: 10.1007/s00216-026-06566-3 (PMC13375963; doi:10.1007/s00216-026-06566-3)
Supplement: Supplementary file 2 — Supplementary file2 (DOCX 1.03 MB) [file 216_2026_6566_MOESM2_ESM.docx]

**SUPPLEMENTARY FIGURES**

**Title: Derivatisation-free FIA-MS/MS assay for rapid simultaneous screening of atypical myopathy biomarkers and acylcarnitine profiles from dried blood spots**

**Authors:** Martina Kadláčková^a,b^, Dana Dobešová^a,b^, Eliška Ivanovová^a,b^, Richard Masař^a,b^, Petr Jahn^c^, Eva Šamonilová^c^, David Friedecký^a,b^, Radana Brumarová^a,b^

^a^ Laboratory for Inherited Metabolic Disorders, Department of Medical Genetics, Faculty of Medicine and Dentistry, Palacký University Olomouc, Hněvotínská 3, 779 00 Olomouc, Czech Republic

^b^ Laboratory for Inherited Metabolic Disorders, Department of Clinical Biochemistry, University Hospital Olomouc, Zdravotníků 248/7, 779 00 Olomouc, Czech Republic

^c^ Equine Clinic, Faculty of Veterinary Medicine, University of Veterinary Sciences Brno, Palackého třída 1946/1, 612 00, Brno-Královo Pole, Czech Republic

**Corresponding author:** Radana Brumarová (radana.brumarova@upol.cz)

**Content**:

**Fig. S1** Chemical structures and selected precursor and product ions used for FIA-MS/MS analysis of acylcarnitines, MCPA-carnitine, and hypoglycin A (HGA). Quantifier and qualifier transitions are indicated for each analyte

**Fig. S2** Evaluation of ion ratios for analytes monitored by FIA-MS/MS. Each point represents an individual measurement obtained from calibration standards, QC samples (LQC and HQC) and DBS samples. The dashed black line indicates the reference ion ratio calculated as the mean value obtained from the six highest calibration levels. Red dashed lines indicate the tolerance limits defined according to Commission Decision 2002/657/EC, and the green area represents the corresponding acceptance interval

**Fig. S3** Calibration curves for HGA, MCPA-carnitine, and seven acylcarnitines obtained by FIA-MS/MS. Curves were constructed from analyte/IS peak area ratios plotted against spiked concentrations in the DBS matrix (endogenous concentration plus spike) using linear regression with 1/x weighting. Points represent mean values from three independent analytical runs; error bars indicate standard deviations (n = 3)

**Fig. S4** Intra-day accuracy and precision (n = 5) on day 1 (A), day 2 (B) and day 3 (C). Purple dotted lines represent validation criteria for LLOQ, orange for LQC, MQC and HQC. Only analytes that did not meet acceptable criteria are indicated


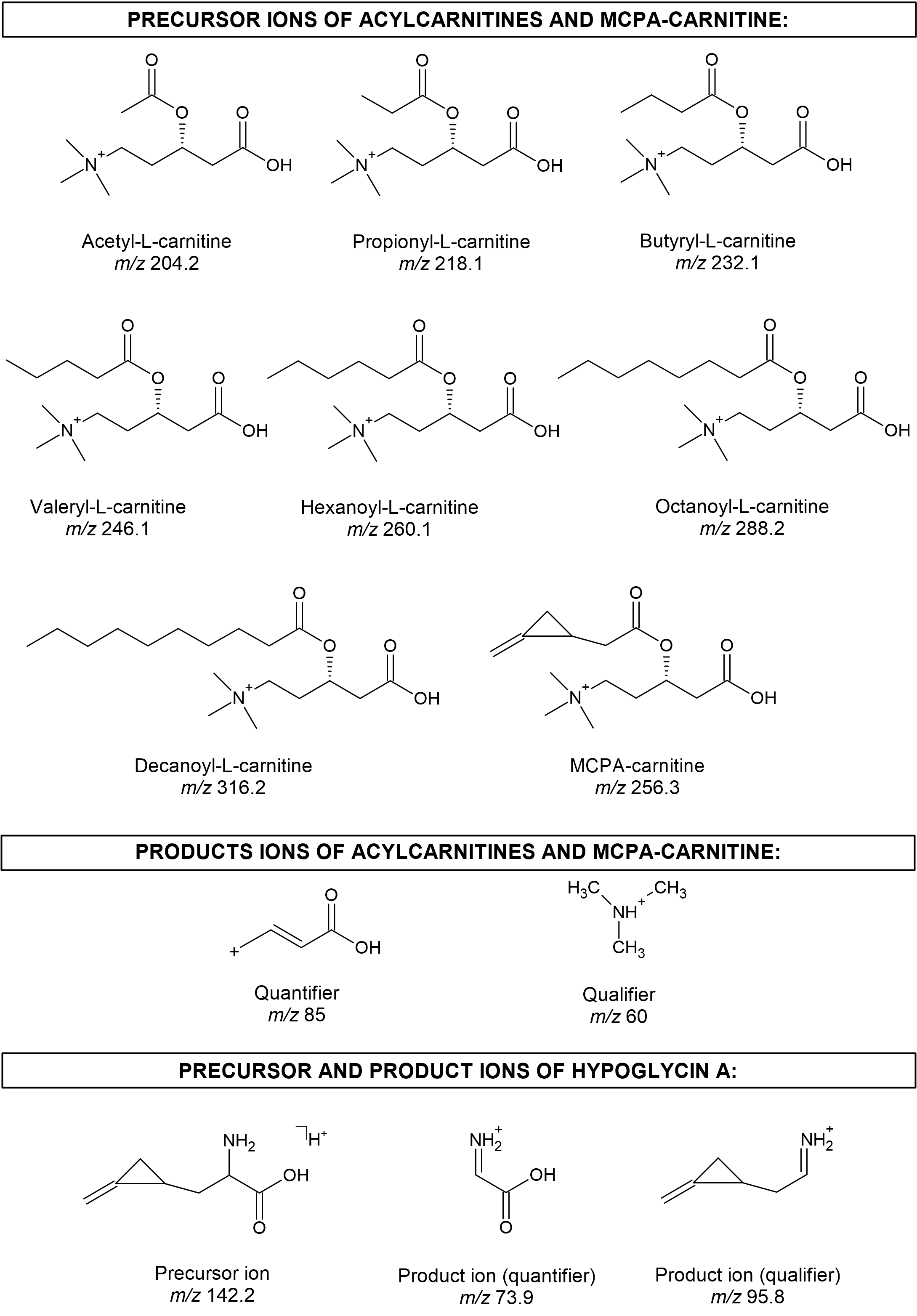


**Fig. S1** Chemical structures and selected precursor and product ions used for FIA-MS/MS analysis of acylcarnitines, MCPA-carnitine, and hypoglycin A (HGA). Quantifier and qualifier transitions are indicated for each analyte


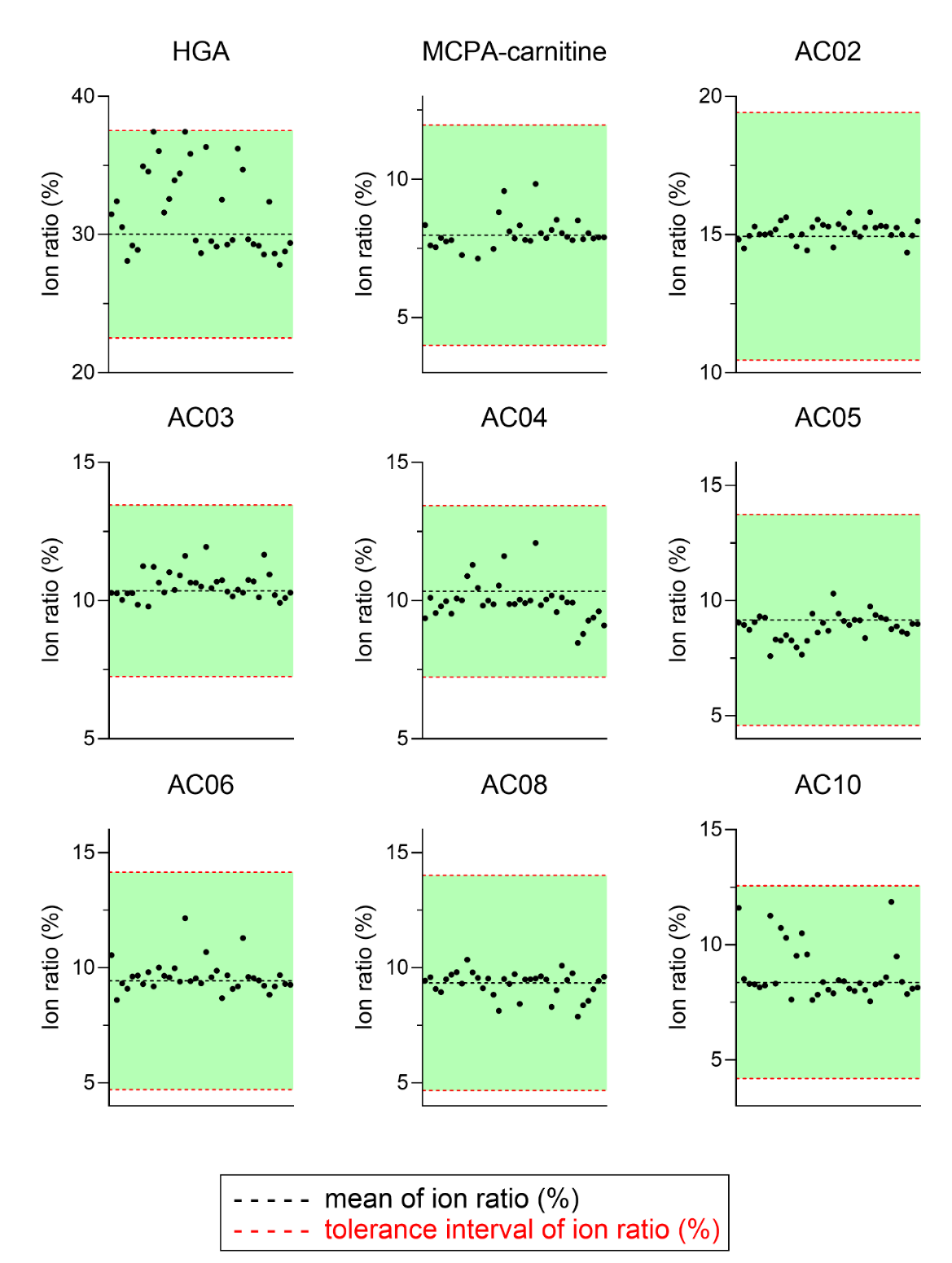


**Fig. S2** Evaluation of ion ratios for analytes monitored by FIA-MS/MS. Each point represents an individual measurement obtained from calibration standards, QC samples (LQC and HQC) and DBS samples. The dashed black line indicates the reference ion ratio calculated as the mean value obtained from the six highest calibration levels. Red dashed lines indicate the tolerance limits defined according to Commission Decision 2002/657/EC, and the green area represents the corresponding acceptance interval


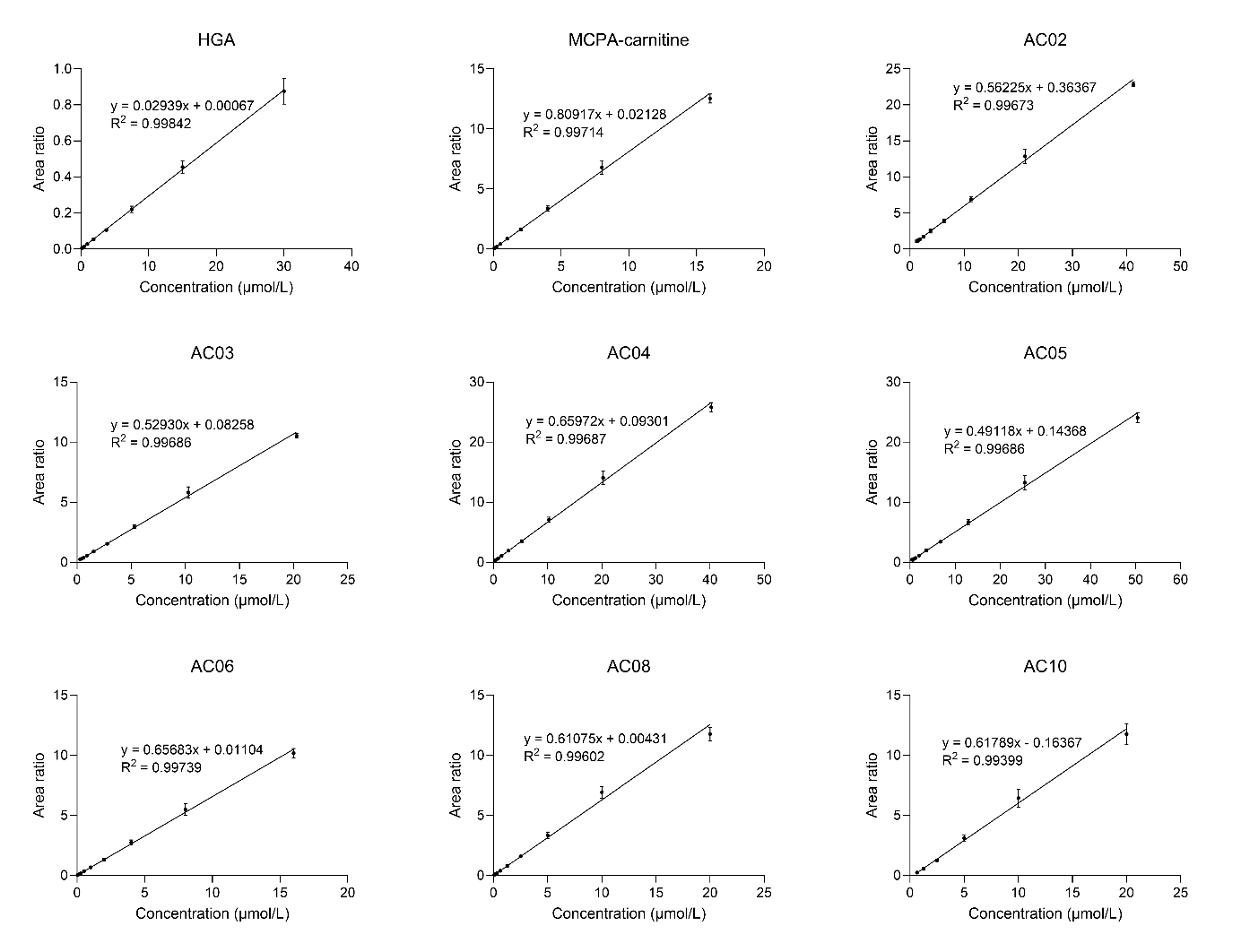


**Fig. S3** Calibration curves for HGA, MCPA-carnitine, and seven acylcarnitines obtained by FIA-MS/MS. Curves were constructed from analyte/IS peak area ratios plotted against spiked concentrations in the DBS matrix (endogenous concentration plus spike) using linear regression with 1/x weighting. Points represent mean values from three independent analytical runs; error bars indicate standard deviations (n = 3)


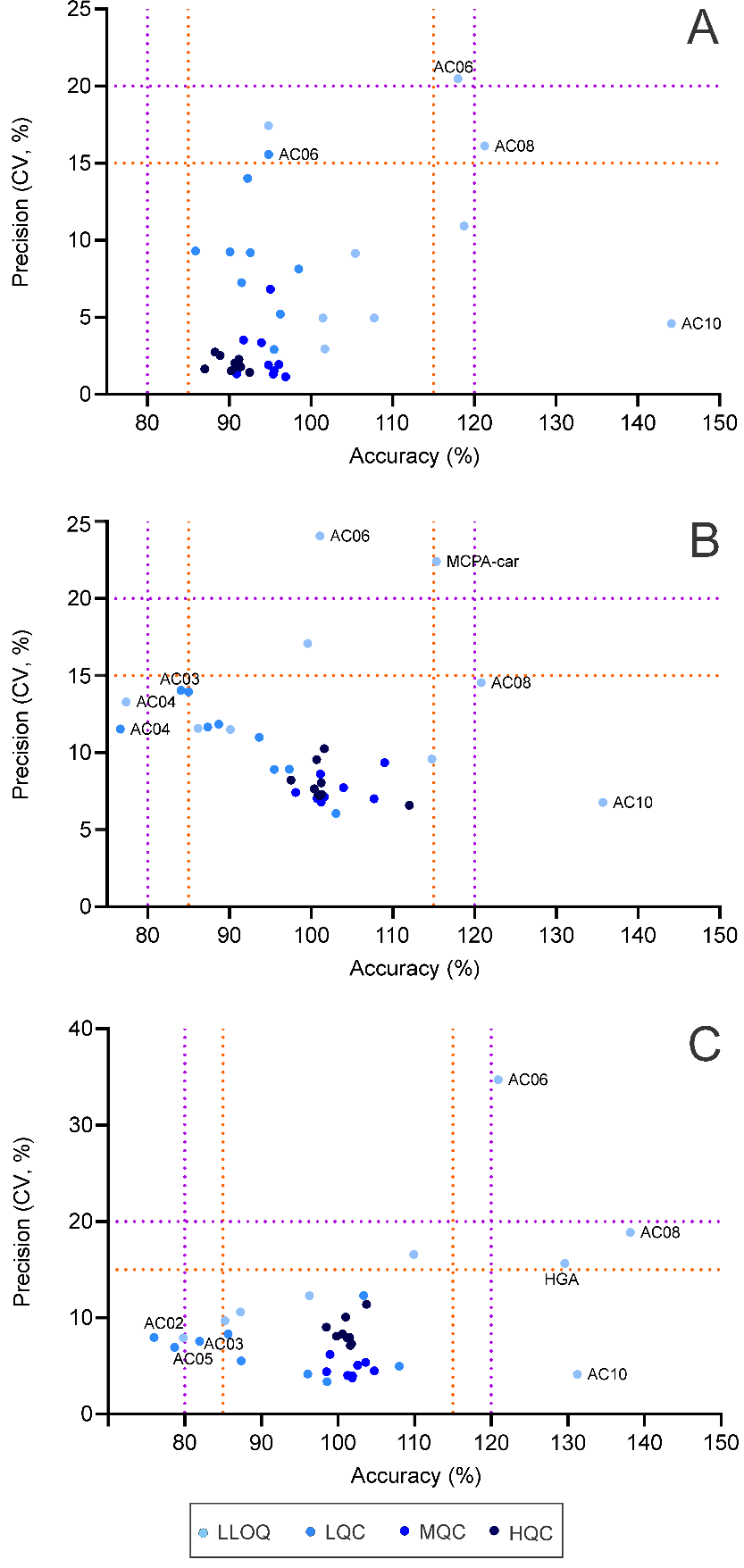


**Fig. S4** Intra-day accuracy and precision (n = 5) on day 1 (A), day 2 (B) and day 3 (C). Purple dotted lines represent validation criteria for LLOQ, orange for LQC, MQC and HQC. Only analytes that did not meet acceptable criteria are indicated
